# Comprehensive behavioral study of C57BL/6.KOR-ApoE^shl^ mice

**DOI:** 10.1515/tnsci-2022-0284

**Published:** 2023-06-30

**Authors:** Hiroshi Ueno, Yu Takahashi, Shinji Murakami, Kenta Wani, Tetsuji Miyazaki, Yosuke Matsumoto, Motoi Okamoto, Takeshi Ishihara

**Affiliations:** Department of Medical Technology, Kawasaki University of Medical Welfare, 288, Matsushima, Kurashiki, Okayama, 701-0193, Japan; Department of Psychiatry, Kawasaki Medical School, Kurashiki, 701-0192, Japan; Department of Neuropsychiatry, Graduate School of Medicine, Dentistry and Pharmaceutical Sciences, Okayama University, Okayama, 700-8558, Japan; Department of Medical Technology, Graduate School of Health Sciences, Okayama University, Okayama, 700-8558, Japan

**Keywords:** apolipoprotein, behavior, behavioral test, central nervous system, mouse

## Abstract

**Background:**

Apolipoprotein E (ApoE) is associated with Alzheimer’s disease (AD) and cognitive dysfunction in elderly individuals. There have been extensive studies on behavioral abnormalities in ApoE-deficient (Apoe^shl^) mice, which have been described as AD mouse models. Spontaneously hyperlipidemic mice were discovered in 1999 as ApoE-deficient mice due to ApoE gene mutations. However, behavioral abnormalities in commercially available Apoe^shl^ mice remain unclear. Accordingly, we aimed to investigate the behavioral abnormalities of Apoe^shl^ mice.

**Results:**

Apoe^shl^ mice showed decreased motor skill learning and increased anxiety-like behavior toward heights. Apoe^shl^ mice did not show abnormal behavior in the Y-maze test, open-field test, light/dark transition test, and passive avoidance test.

**Conclusion:**

Our findings suggest the utility of Apoe^shl^ mice in investigating the function of ApoE in the central nervous system.

## 1 Background

Apolipoprotein E (ApoE) is a protein with a molecular weight of 34,200 Da that consists of 299 amino acids. In humans, ApoE is the major cholesterol carrier involved in lipid transport. Furthermore, it is involved in lipid metabolism, including cholesterol transport and lipoprotein metabolism as well as activation of lipid-metabolizing enzymes (liver lipase, lipoprotein lipase, lecithin/cholesterol acyltransferase, etc.). In humans, the apolipoprotein E gene (APOE) has three major polymorphic allelic variants. Moreover, the resulting apoE isoforms (apoE2, apoE3, and apoE4) only differ by single amino acids. The major isoforms (ApoE2, ApoE3, ApoE4) differ by two amino acid substitutions at positions 112 and 158; specifically, ApoE2 is Cys/Cys, ApoE3 is Cys/Arg, and ApoE4 is Arg/Arg [1]. These small differences significantly influence their biochemical properties and function [2,3].

In addition to lipid metabolism, ApoE and ApoE isoforms are involved in the maintenance of normal brain function [4]. Moreover, ApoE is expressed in the central and peripheral nervous systems [5], where it is mainly produced and secreted by astrocytes [6]. Since ApoE is a major cholesterol required for neural activity and brain damage repair, it has been shown to be involved in debris removal from damaged cells and stimulation of nerve cell regeneration [7].

There have been studies on behavioral abnormalities in ApoE-deficient mice. ApoE-deficient mice show reduced contact with the herd during sleep and reduced motor activity in new environments as well as learning and memory deficits [8]. ApoE deficiency causes learning disabilities in behavioral tasks related to hippocampal function in mice [9,10]. These behavioral abnormalities are consistent with cognitive impairment and memory loss, which are the earliest clinical manifestations of neurodegenerative diseases such as Alzheimer’s disease (AD). Therefore, ApoE-deficient mice could be used as AD models [11]. In addition, ApoE-deficient mice exhibit cholinergic dysfunction, tau hyperphosphorylation [12,13], and memory impairment in complex tasks related to hippocampal function [14], which are observed in AD.

Worldwide, studies have used ApoE-deficient mice as an AD model. ApoE-deficient mice are commercially available as B6.129P2-Apoe^tm1Unc^ mice and are homozygous to the mutant Apo-E^tm1Unc^. ApoE-deficient mice are deficient in ApoE, a ligand for the uptake of chylomicrons into the liver via low-density lipoprotein (LDL) and remnant receptors [15]. As a result, cholesterol-rich remnant particles increase, resulting in the formation of atherosclerotic lesions in the proximal aorta [16]. ApoE-deficient mice are also considered one of the models of human arteriosclerosis because the onset site and progression of the lesion are similar to those of humans [17].

On the other hand, Matsushima et al. have discovered spontaneously hyperlipidemic (SHL) mice in the process of producing from wild mice to inbred mice [18]. This mouse is a naturally mutated mouse. Spontaneous hyperlipidemia (Apoe^shl^) mice have been shown to develop due to apolipoprotein E deficiency due to mutations in the apolipoprotein E gene [19]. Then, they introduced Apoe^shl^, which is the causative gene of hyperlipidemia, into the genetic background of the C57BL/6 strain of the arteriosclerosis susceptibility strain. Subsequently, they produced B6.KOR/StmSlc-Apoe^shl^ (B6.SHL) mice [19], which are now commercially available. ApoE-deficient mice are the most common AD model mice; however, they require high cost and time to obtain from the Jackson Laboratory in the United States. In addition, experiments using ApoE-deficient mice are resource intensive and energy intensive, given the regulatory procedures for genetically modified organisms. Contrastingly, SHL mice available from Japan SLC have spontaneous APOE mutations, and therefore, these restrictions do not apply. Given the high cost of ApoE-deficient mice and the complexity of maintaining transgenic animals, Apoe^shl^ mice are more natural, allow better elucidation of temporal changes in pathology, and could be more useful [19]. Compared with ApoE-deficient mice, spontaneous Apoe^shl^ mice could be more useful as a model for hyperlipidemia, arteriosclerosis, and AD. However, behavioral and central nervous system abnormalities in Apoe^shl^ mice remain unclear.

This study aimed to clarify behavioral abnormalities in Apoe^shl^ mice. Specifically, we aimed to elucidate the effects of ApoE on behavior and the central nervous system, which could demonstrate the utility of Apoe^shl^ mice.

## 2 Methods

### 2.1 Animals

We purchased 8-week-old C57BL/6.KOR-ApoE^shl^ and wild-type C57BL/6N male mice from Japan SLC (Shizuoka, Japan). Subsequently, they were housed in cages (five animals per cage) with food and water provided ad libitum under a 12 h light/dark cycle at 23–26°C. Since behavioral diversity is partially sex dependent and this study did not seek to compare sex differences in behavior, we only included male mice.

### 2.2 Behavioral tests

All behavioral tests were conducted in behavioral testing rooms between 09:00 and 16:00. After the tests, the equipment and toys were cleaned using 70% ethanol and super-hypochlorous water to prevent artifacts caused by lingering olfactory cues. Behavioral tests were performed in naïve mice following the test order described below. The same animals were used for different behavioral tests [20]. After the experiments, the animals were sacrificed through CO_2_ inhalation.

### 2.3 Wire hang test

In the wire hang test, each mouse was placed on a wire mesh, which was then inverted and waved gently so that the mouse gripped the wire. Subsequently, latency to fall was recorded. A wire hang test apparatus (O’Hara & Co., Tokyo, Japan) was used.

### 2.4 Grip strength test

Neuromuscular strength was examined using the grip strength test. Forelimb strength was measured using a grip strength meter. Mice were lifted and held by the tail such that their forepaws could grasp a wire grid; subsequently, they were gently pulled back until they released the grid. The peak force applied by the forelimbs was recorded in Newtons (cN).

### 2.5 Rotarod test

Motor coordination and balance were tested using the rotarod test as previously described [21]. This test, which uses an accelerating rotarod (RTR-M5; Melquest, Toyama, Japan), was performed by placing the mouse on rotating drums (3.9-cm diameter) to measure the time it took to maintain balance on this rod. The rotarod speed was accelerated from 4 to 40 rpm over 5 min. The intertrial interval for this test was 20 min. All mice were subjected to the test without previous training.

### 2.6 Cotton bud biting test

Aggressive behavior was examined using the cotton bud biting test as previously described [22]. The mice were placed on a hand and a sterilized cotton bud was held close to their faces. Biting on the cotton bud was considered aggressive behavior. The mice were tested ten times. We analyzed the total number of biting attacks.

### 2.7 Hot plate test

The hot plate test was used to evaluate nociception (sensitivity to a painful stimulus) [23]. Mice were placed on a plate heated to 55.0 ± 0.3°C and the latency to the first paw response was recorded. Paw responses comprised foot shakes or paw licks. A 30-s latency period was defined as complete analgesia and it was used as the cutoff time for preventing tissue injury.

### 2.8 Elevated plus maze test

We examined anxiety-like behavior using the elevated plus maze [24]. The apparatus comprised two open arms (8 cm × 25 cm) and two closed arms of the same size, with 30-cm-high transparent walls. The arms were constructed from white plastic plates and elevated to 40 cm above the floor. Arms of the same type were located opposite each other. Each mouse was placed in the central square of the maze, facing a closed arm, and allowed to freely move between the four arms for 6 min. The mice were video-recorded; moreover, we analyzed the number of arm entries, distance traveled (m), and time spent in the open arms using video tracking software (ANY-MAZE, Stoelting Co., Wood Dale, IL, USA).

### 2.9 Light/dark transition test

Light/dark transition test was examined as previously described [25,26]. The apparatus comprised an acrylic cage (22 cm × 44 cm × 40 cm) divided into two equally sized sections by a partition with a door. One chamber had white acrylic walls and was brightly illuminated (200 lx) by lights above the chamber ceiling. The other chamber had black acrylic walls and was dark (50 lx). Both chambers had a white plastic floor. The mice were placed into the dark chamber and allowed to freely move between both chambers for 6 min with the door open. The distance traveled (m), total number of transitions, and time spent in the light chamber (s) were analyzed using the ANY-MAZE software.

### 2.10 Open-field test

The open-field test was used to examine exploratory behavior, anxiety-like behavior, and general locomotor activity [24]. Each mouse was placed in the center of an apparatus, which comprised a square area surrounded by walls (45 cm × 45 cm × 40 cm). The distance traveled (m), number of entries into the central area, and time spent in the central area (s) were recorded. We defined the central area as the middle 20 cm × 20 cm field portion. The test chamber was illuminated at 100 lx. Data were collected over 30 min. Data analysis was performed using ANY-MAZE software.

### 2.11 Y-maze test

We measured spatial working memory using a Y-maze apparatus (arm length: 40 cm, arm bottom width: 3 cm, upper arm width: 10 cm, wall height: 12 cm) [24]. The mice were placed at the center of the Y-maze for 6 min. Visual cues were placed around the maze in the testing room and maintained there throughout the tests. Mice were tested without previous exposure or habituation to the maze. The total distance traveled (m), number of entries, and number of alternations were recorded and analyzed using ANY-MAZE software.

### 2.12 Tail suspension test

Depressive-like behavior was examined using the tail suspension test [24]. Each mouse was suspended by the tail 60 cm above the floor in a white plastic chamber using adhesive tape placed <1 cm from the tail tip. The resultant behavior was recorded for 6 min. Images were captured using a video camera and immobility time was measured. The “immobile period” was defined as the interval when the animals stopped struggling for ≥1 s. Data acquisition and analysis were performed using ANY-MAZE software.

### 2.13 Porsolt forced swim test

Depressive-like behavior was also examined using the Porsolt forced swim test. The apparatus comprised four Plexiglas cylinders (20-cm height × 10-cm diameter). The cylinders were filled with water (23°C) to a depth of 7.5 cm as previously described [27,28]. The mice were placed in the cylinders for 6 min and their behavior was recorded. Similar to the tail suspension test, immobility time was evaluated using ANY-MAZE software.

### 2.14 Passive avoidance test

A two-compartment step-through passive avoidance apparatus (MPB-M020; Melquest) was used as previously described [29]. The apparatus is divided by a wall with a guillotine door into a bright (9.0 cm × 18.0 cm × 14.5 cm) and dark (18.0 cm × 18.0 cm × 14.5 cm) compartment. The bright compartment was illuminated using fluorescent light (200 lx). The mice were placed in the bright compartment and allowed to explore for 20 s; subsequently, the guillotine door was raised to allow the mice to enter the dark compartment and closed upon entry. Next, an electrical foot shock (0.5 mA) was delivered for 3 s. Test sessions were performed 24 h after training sessions. The latency to enter the dark compartment was recorded for up to 180 s.

### 2.15 Statistical analyses

Data were analyzed using two-way repeated-measures analysis of variance (ANOVA), Fisher’s least significant difference test, or Student’s *t*-tests. Statistical significance was set at *p* < 0.05. Data are presented as box plots.


**Ethical approval:** The research related to animals’ use has been complied with all the relevant national regulations and institutional policies for the care and use of animals. All animal experiments were performed following the ARRIVE guidelines (http://www.nc3rs.org.uk/arrive-guidelines) and the U.S. National Institutes of Health (NIH) Guide for the Care and Use of Laboratory Animals (NIH Publication No. 80-23, revised in 1996); moreover, they were approved by the Committee for Animal Experiments at Kawasaki Medical School Advanced Research Centre. All efforts were made to minimize the number and suffering of animals used. The required sample size was calculated using a power analysis.

## 3 Results

### 3.1 General characterization of Apoe^shl^ mice

**Figure 1:**
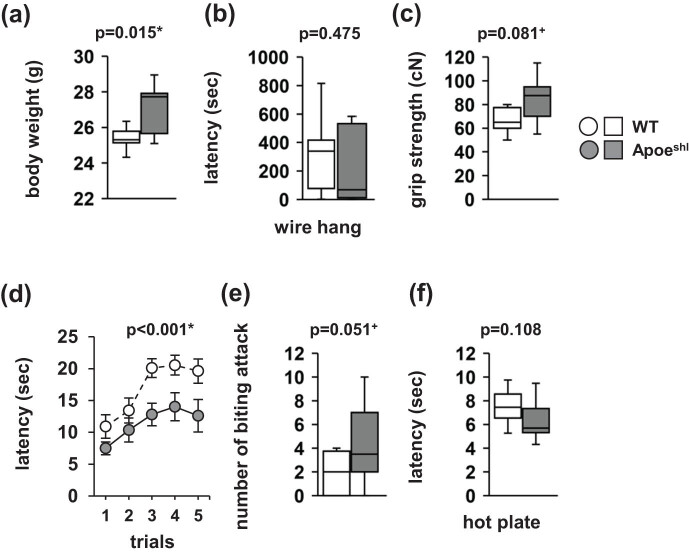
Normal physical characteristics of Apoe^shl^ mice. (a) Body weight. (b) Latency to fall in the wire hang test. (c) Grip strength. (d) Latency to fall in the rotarod test. (e) Number of times biting on the cotton bud. (f) Hot plate test. Data are presented as box plots (a–c, e, f) or the mean ± standard error (d). Statistical significance is indicated by asterisks: *p < 0.05, ^+^ *p* < 0.1. The *p*-values were calculated using Student’s *t*-test (a–c, e, f) or two-way repeated-measures ANOVA (d). (a–f) Wild-type (WT): *n* = 10, Apoe^shl^: *n* = 10.

Apoe^shl^ mice appeared healthy with no obvious differences in physical characteristics compared with wild-type mice. Apoe^shl^ mice had significantly higher body weight than wild-type mice (Figure 1a, *p* = 0.015). There were no significant between-group differences in the latency to fall in the wire hang test (Figure 1b, *p* = 0.475) as well as in grip strength (Figure 1c, *p* = 0.081). As shown in Figure 1d, Apoe^shl^ mice showed decreased latency to fall in the rotarod test (*F*_1,90_ = 21.652, *p* < 0.001). In the cotton bud biting test, there were no significant between-group differences in the number of biting attacks (Figure 1e, *p* = 0.051). In the hot plate test, there were no significant between-group differences in the pain threshold (Figure 1f, *p* = 0.108).

### 3.2 Apoe^shl^ mice showed increased anxiety-like behavior in the elevated plus-maze test

**Figure 2:**
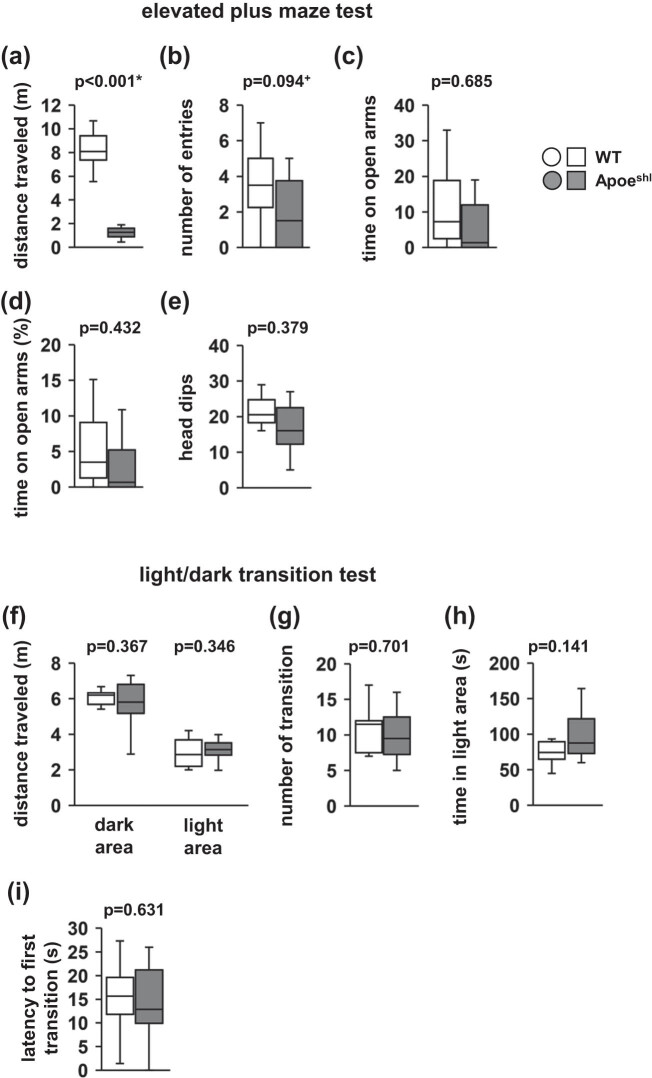
Performance of Apoe^shl^ mice in the elevated plus maze test. Elevated plus-maze test: total distance traveled (a), number of total entries into open arms (b), time spent in the open arms (c), percentage of open arm time (d), and head dips (e). Light/dark transition test: total distance traveled (f), number of light/dark transitions (g), time spent in the light area (h), and latency to first transition into the light area (i). Data are presented as box plots (a–i). Statistical significance is represented by asterisks: *p < 0.05, ^+^ *p* < 0.1. The *p*-values were calculated using Student’s *t*-test (a–e and g–i) or two-way ANOVA (f). (a–i) Wild-type (WT): *n* = 10, Apoe^shl^: *n* = 10.

Anxiety-like behavior was evaluated using the elevated plus maze. Apoe^shl^ mice showed a significantly lower total distance traveled than wild-type mice (Figure 2a, *p* < 0.001). There were no between-group differences in the total number of entries into open arms (Figure 2b, *p* = 0.094), time spent in the open arms (Figure 2c, *p* = 0.685), percentage of open arm time (Figure 2d, *p* = 0.432), and head dips (Figure 2e, *p* = 0.379).

### 3.3 Unaltered light/dark transition in Apoe^shl^ mice

In the light/dark transition test, there were no significant between-group differences in the distance traveled (Figure 2f, *F*_1,36_ = 0.011, *p* = 0.915), number of transitions between light/dark compartments (Figure 2g, *p* = 0.701), time spent in the light compartment (Figure 2h, *p* = 0.141), and latency to first transition into the light area (Figure 2i, *p* = 0.631).

### 3.4 Performance of Apoe^shl^ mice in the open-field test

**Figure 3:**
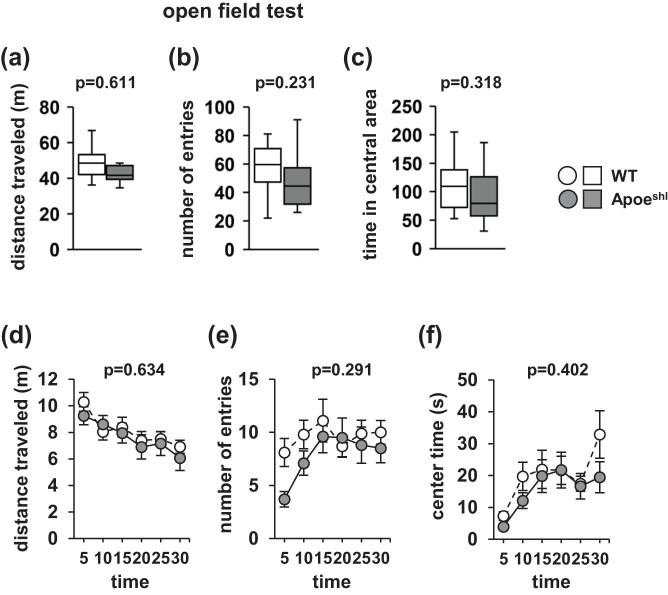
Performance of Apoe^shl^ mice in the open-field test. Graphs showing the total distance traveled (a), number of total entries into the central area (b), and total time spent in the central area (c) in the open-field test are presented. Graphs showing the distance traveled (d), number of entries into the central area (e), and time spent in the central area (f) in each of the 5-min tests are also depicted. Data are presented as box plots (a–c) or the mean ± standard error (d–f). Statistical significance is represented by asterisks: *p < 0.05, ^+^ *p* < 0.1. The *p*-values were calculated using Student’s *t*-test (a–c) or two-way repeated-measures ANOVA (d–f). (a–f) Wild-type (WT): *n* = 10, Apoe^shl^: *n* = 10.

In the open-field test, there were no significant between-group differences in the total distance traveled (Figure 3a, *p* = 0.611), number of entries into the central area (Figure 3b, *p* = 0.231), or time spent in the central area (Figure 3c, *p* = 0.315). There were no significant between-group differences in the distance traveled (Figure 3d, *F*_1,108_ = 0.687, *p* = 0.634), number of entries into the central area (Figure 3e, *F*_1,108_ = 1.254, *p* = 0.291), and time spent in the central area (Figure 3f, *F*_1,108_ = 1.034, *p* = 0.402) in each 5-min period.

### 3.5 Performance of Apoe^shl^ mice in the Y-maze test

**Figure 4:**
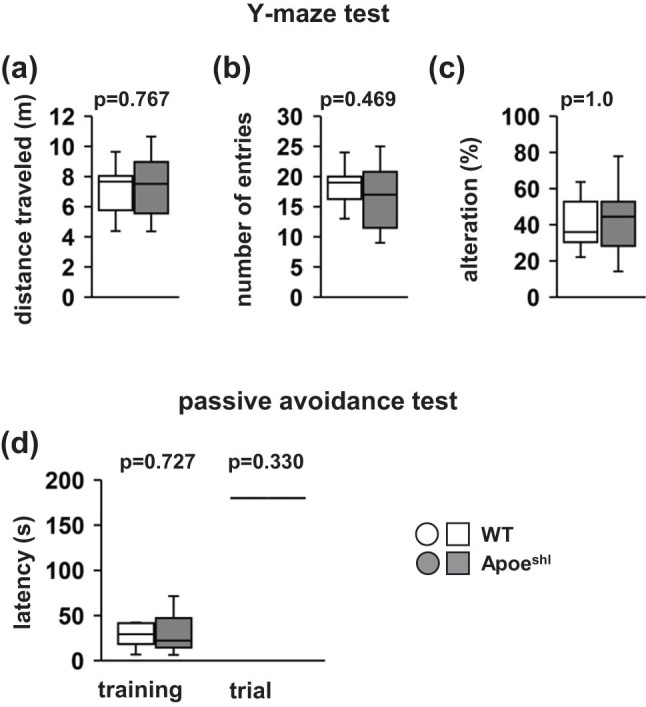
Performance of Apoe^shl^ mice in the Y-maze test and passive avoidance test. Y-maze test: total distance traveled (a), total number of arm entries (b), and percentage of alternations (c). Passive avoidance test: the escape latencies during the training session and retention test (d). In the retention test conducted 24 h after training, the maximum latency was set as 180 s. Data are presented as box plots (a–d). Statistical significance is represented by asterisks: *p < 0.05, ^+^ *p* < 0.1. The *p*-values were calculated using Student’s *t*-test (a–c) or two-way ANOVA (d). (a–d) Wild-type (WT): *n* = 10, Apoe^shl^: *n* = 10.

In the Y-maze test, there were no significant between-group differences in the total distance traveled (Figure 4a, *p* = 0.767), number of arm entries (Figure 4b, *p* = 0.469), and percentage of alternations in the total number of entries (Figure 4c, *p* = 0.750).

### 3.6 Normal learning and memory of Apoe^shl^ mice in the passive avoidance test

In the step-through passive avoidance test, there was no significant between-group difference in the latency to enter the dark compartment throughout the conditioning session (Figure 4d, *F*_1,36_ = 0.009, *p* = 0.924). At 24 h after the conditioning session, the step-through latency was not lower in Apoe^shl^ mice than in wild-type mice during the retention trials (Figure 4d).

### 3.7 Normal immobility of Apoe^shl^ mice in tests for depression-like behavior

**Figure 5:**
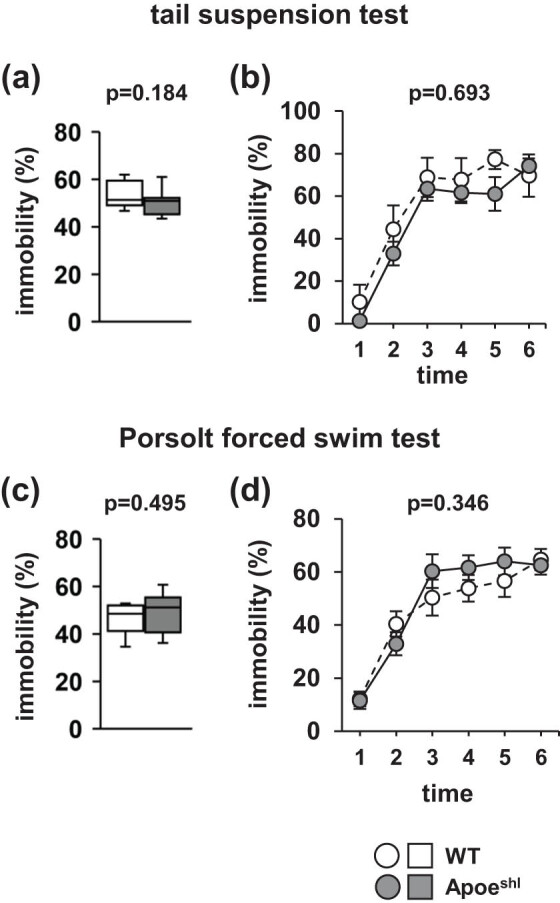
Depressive-like behavior of Apoe^shl^ mice. Tail-suspension test: the proportion of total time spent immobile (a) and the proportion of time spent immobile in each 1-min period (b). Porsolt forced swim test: the proportion of total time spent immobile (c) and the proportion of time spent immobile in each 1-min period (d). Data are presented as box plots (a, c) or the mean ± standard error (b, d). Statistical significance is represented by asterisks: *p < 0.05, ^+^ *p* < 0.1. The *p*-values were calculated using Student’s *t*-test (a, c) or two-way repeated-measures ANOVA (b, d). (a–d) Wild-type (WT): *n* = 10, Apoe^shl^: *n* = 10.

In the tail-suspension test, there were no significant between-group differences in the total immobile time (Figure 5a, *p* = 0.184) and percentage of time spent immobile in each 1-min period (Figure 5b, *F*_1,108_ = 0.610, *p* = 0.693). In the Porsolt forced swim test, there were no significant between-group differences in the total immobile time (Figure 5c, *p* = 0.495) and the percentage of time spent immobile in each 1-min period (Figure 5d, *F*_1,108_ = 1.139, *p* = 0.346).

## 4 Discussion

Our findings showed that Apoe^shl^ mice presented abnormal behavior compared with wild-type mice. Apoe^shl^ mice showed decreased motor skill learning and increased anxiety-like behavior toward heights. Compared with wild-type mice, Apoe^shl^ mice lacked clear changes compared with many other behavioral tests.

In this study, 10-week-old Apoe^shl^ mice were significantly heavier than wild-type mice, which is consistent with previous reports of increased body weight from the age of 2 months in Apoe^shl^ mice compared with wild-type mice [30]. ApoE deficiency and abnormalities cause hyperlipoproteinemia type III, which is characterized by early atherosclerosis and cholesterol accumulation in the blood. ApoE-deficient mice develop lesions of severe hypercholesterolemia and atherosclerosis, which may resemble those in humans [15,31]. Weight gain in Apoe^shl^ mice could be attributed to abnormal lipid metabolism.

There was no significant between-group difference in the wire hang test; however, Apoe^shl^ mice tended to show increased forelimb grip strength. Muscle strength assessment is crucial for assessing neuromuscular disorders using rodent models [32]. The ε4 allele of the APOE gene (APOE4) is related to muscle weakness [33]. Further research is warranted to elucidate the mechanism underlying the effect of ApoE deficiency on muscle strength.

Compared with wild-type mice, Apoe^shl^ mice showed a marked decrease in motor function in the rotarod test. Normal motor function has been observed in 12-month-old ApoE-deficient mice [8]. However, APOE4 is associated with a rapid decline in motor function in elderly individuals [33]. This association does not change after adjusting for body composition, cognitive status, vascular risk factors, and disease. The mechanism underlying the relationship between decreased motor function and ApoE remains unclear; however, ApoE deficiency may damage the motor system. It is important to study motor dysfunction in young mice because motor dysfunction develops earlier than cognitive deficits [34]. Also, the decline in exercise performance in the rotarod test in Apoe^shl^ mice may be due to increased body weight. Behavioral tests to measure motor function that can rule out the weight gain factor should be considered.

Apoe^shl^ mice tended to be more aggressive than wild-type mice. APOE4 is associated with aggressive behavior in elderly individuals [35]. Contrastingly, ApoE-deficient mice present less aggressive behavior than wild-type mice [36]. Although the mechanism underlying enhanced aggressive behavior in Apoe^shl^ mice remains unclear, our findings demonstrate that Apoe^shl^ mice are more suitable as AD mouse models than ApoE-deficient mice.

Compared with wild-type mice, Apoe^shl^ mice showed reduced distance traveled in the elevated plus maze test. Apoe^shl^ mice showed a tendency of a decreased number of entries into the open arm. Compared with wild-type mice, Apoe^shl^ mice showed increased anxiety-like behavior; however, they lacked behavioral abnormalities in the light/dark transition test and open-field test. There are several types of anxiety-like behavior, including anxieties about high places, bright places, and large objects [37,38]. Our findings demonstrated that Apoe^shl^ mice have increased anxiety regarding high altitude. Anxiety is the most common symptom in patients with AD with an onset age <65 years [39]. Moreover, compared with wild-type mice, ApoE-deficient mice showed anxiety-like behavior in the elevated plus maze test [40]. However, this anxiety-like behavior was not observed in 3-month-old ApoE-deficient mice. ApoE-deficient mice have increased basal adrenal corticosterone levels and abnormally increased plasma corticosterone levels after anxiety assessment using the elevated plus maze test [40]. Further research is warranted to determine whether Apoe^shl^ mice have increased basal adrenal corticosterone levels. In addition, 2-month-old Apoe^shl^ mice showed increased anxiety-like behavior compared with ApoE-deficient mice, which better reflects the onset of juvenile AD compared with ApoE-deficient mice.

We observed no significant between-group differences in the Y-maze and passive avoidance tests. A previous study reported that 12-month-old ApoE-deficient mice show decreased cognitive function in the Y-maze test and memory impairment in the passive avoidance test [8]. However, ApoE-deficient mice have shown good performance in spatial tasks [41,42]. These discrepancies could be attributed to mouse age, environmental factors, compensation with other proteins, genetic background, and adopted protocols and behavioral tasks [43,44]. Behavioral tests at various ages are required to clarify the cognitive function and memory ability in Apoe^shl^ mice. Nonetheless, the findings demonstrated that 2-month-old Apoe^shl^ mice showed no decline in cognitive function or memory retention.

Similar to ApoE-deficient mice, Apoe^shl^ mice did not exhibit increased depressive-like behavior in tail suspension and forced swim tests. In the early stages, patients with AD may exhibit memory impairment and depressive-like behavior [45]. ApoE is associated with depression [46]. Further research on depressive-like behavior at different ages of Apoe^shl^ mice is warranted.

ApoE is expressed and secreted by multiple cell types in both the central and peripheral nervous systems, including astrocytes, microglia, vascular mural cells, hepatocytes, and macrophages [47,48,49]. Several studies have suggested that ApoE deficiency increases neuronal sensitivity to oxidative damage [50,51]. ApoE-deficient mice develop age-dependent synaptic loss [9,12]. Notably, Apoe^shl^ mice have increased plasma lipid levels [18], which can independently cause synaptic dysfunction and cognitive impairment [52]. Hypertriglyceridemia in mice causes cognitive dysfunction [53]. Another mechanism through which loss of ApoE can cause abnormal behavior is its effect on the vascular system. Vascular dysfunction and atherosclerosis occur early in ApoE-deficient mice, which results in decreased cerebral blood flow and autonomic dysregulation of the cerebrovascular system [15,54]. Further research is warranted to investigate the mechanism underlying abnormal behavior in Apoe^shl^ mice. In addition, it should be performed in older animals.

Unlike humans, mice express only one type of ApoE [55,56]. Therefore, the utility of Apoe^shl^ mouse as a human disease model remains unclear.

Compared with male ApoE-deficient mice, female ApoE-deficient mice are more susceptible to cognitive dysfunction [30,57]. In fact, women have a higher risk of AD than men [58,59]. We performed experiments on male Apoe^shl^ mice; accordingly, similar behavioral tests on female Apoe^shl^ mice are warranted.

## 5 Conclusions

Apoe^shl^ mice showed reduced motor skill function and increased anxiety-like behavior. However, they lacked abnormal behavior in the Y-maze test, open-field test, light/dark transition test, and passive avoidance test. Our findings demonstrated that Apoe^shl^ mice are useful as a model for investigating ApoE function in hyperlipidemia and in the central nervous system.

## List of Abbreviations

AD: Alzheimer’s disease
ANOVA: Analysis of variance
Apo: Apolipoprotein.
